# Effect of Falcaria Vulgaris on Milk Production Parameters in Female Rats’ Mammary Glands 

**Published:** 2018-12

**Authors:** Mohammad Reza Salahshoor, Mohammad Mehdi Mohammadi, Shiva Roshankhah, Cyrus Jalili

**Affiliations:** 1 Department of Anatomical Sciences, Medical School, Kermanshah University of Medical Sciences, Kermanshah, Iran; 2 School of Nursing and Midwifery, Kermanshah University of Medical Sciences, Kermanshah, Iran

**Keywords:** Milk, Mammary Glands, Animal

## Abstract

**Objective:** Falcaria Vulgaris (F.Vulgaris) is a multipurpose herb in traditional medicine, containing essential amino acids, vitamins and numerous nutrients. The aim of this study was to determine the effects of F.Vulgaris on the on the milk production parameters in rats.

**Materials and methods:** In this experimental study, 32 wistar female rats were equally divided into 4 groups; group1, control group and group2-4, experimental groups by various doses of F.Vulgaris (20, 50, 100 mg/kg) injected interaperitoneally once a day for 21 consecutive days. The milk production parameters including, diameter and number of alveoli, prolactin hormone level and receptor prolactin (PRLR) gene expression in mammillary gland tissue were measured and compared.

**Results:** The results indicated that F.Vulgaris significantly increased diameter and number of alveoli, prolactin hormone level and PRLR gene expression compared to control group (p < 0.05).

**Conclusion:** F.Vulgaris extract has positive effects on the milk production parameters in mammary glands.

## Introduction

Postpartum child care and feeding is one of the issues of paramount importance to anyone. In the meantime, breastfeeding is the best nutrition for a child up to the age of 6 months, which in addition to satisfying physical and physiological needs, complements the needs of the child and mother (1). Breast milk can prevent some diseases in the first year of life (2). Despite the fact that mothers are more interested in breastfeeding, less than 50% of mothers are able to continue breastfeeding during breastfeeding period, and mothers encounter early breastfeeding discontinuation in most cases (3). The importance of breastfeeding in the first 2 years postpartum and the problems that are taking place for mothers in this area show the need for new research into the availability and production of drugs increasing breast milk production (4). However, according to the World Health Organization, 1.5 million infant deaths occur each year due to lack of breastfeeding denial or lack of getting enough milk (5). The level of prolactin secretion increases physiologically during pregnancy and postpartum during lactation in such way that prolactin concentration is 20 times higher than the normal level during pregnancy (6). It has been known that prolactin plays different roles, such as the sustainability of the secretion of the mammary gland, its concomitant action with androgens, and its effect on the metabolism of androgens (7). Treatments are required to improve breastfeeding and enhance mothers' milk production and chemical drugs are often used to solve this problem. Research on chemical drugs has been used as breast milk increasing drugs, but these drugs are not widely used due to their side effects (8). It seems that one of the ways to increase breast milk is to use herbal medicines. The Falcaria vulgaris plant, with the local name of Ghazzyaghi / paghaza, belongs to the Umbelliferae family that grows near the farms and is used as a vegetable in some parts of Iran (9). This plant has traditionally been used to improve skin ulcers, gastric disorders, including gastric ulcer, liver disease, and kidney and bladder stones in the west of the country. Phytochemical studies of the plant have shown the presence of tannin and saponin (10). In traditional medicine, this plant is also used to increase breast milk supply (11). It also contains vitamin C, phytosterol, protein and starchy materials and is used to treat skin ulcers, like many antibiotics (12). The results of HPLC analysis showed that the antioxidant and anti-microbial compounds of F. vulgaris contain the highest concentrations of carvacrol and fumaric acid as 119 and 966 mg / kg, respectively (13). The main elements of different parts of the plant have shown antioxidant activity and the neutralizing properties of free radicals (14). Considering the different properties of this plant and the fact that no studies have been carried out on the effects of F. vulgaris on mammary glands and milk production so far, we designed this study to investigate the effect of F. Vulgaris on milk production parameters of rats.

## Materials and methods

***Animals:*** In this experimental study, 32 adult female wistar race rats (2-3 months) with a weight range of 180-220 g were purchased from Razi Institute of Iran and they were used in the study. After fertilization of female rats by male, within 20 days of pregnancy, on average, every mother has 10 Newborn. For one week before the start of the research, they were kept in animal house of Kermanshah University of Medical Sciences under laboratory conditions and at temperatures 20 ± 2 ºC, 12 hours in lighting and 12 hours in darkness conditions. To keep the rats, standard cages in medical school that there are 8 mice in each cage were used (15). 

***Extract preparation*** ***:*** F.vulgaris plant was obtained from a local store from which impurities were removed. After endorsement by a botanist, the plant was cleaned. The leaves and stems were dehydrated in shadow for 5 days and ground by a grinder. 100 gr of the power was added to 70% ethanol. The acquired solution was reserved in hot water bath (36 ºC) in dark condition. Then, the solution was progressively poured on Buchner funnel filter paper and cleans by vacuum pump. It was then transferred to rotary device to get the extra solvent. The isolation process was continued until the concentrated extract was found. The extract was dissolved in distilled water and administered intraperitoneally per a kilogram of animal’s weight (9). 

***Experimental design:*** The rats were randomly divided into four groups (n = 8). Group 1, control group, received normal saline, other group’s equivalent; groups 2 to 4, experimental groups (F.vulgaris groups) were given 20, 50 and 100 mg/kg F.vulgaris respectively. In groups 2 to 4 F.vulgaris was injected interaperitoneally once a day for 28 consecutive days. Rats in groups 6-8, Four weeks after induction of diabetes, received F.vulgaris daily for 21 consecutive days. The same volume of saline was injected in group1 (3, 9 and 10).

***Radio Immune Assay (RIA):*** The animals were anesthetized by injection of 2% Xylazine and 10% ketamine, and blood was collected from their hearts by syringe. The blood of animals was slowly poured into the test tubes and kept at laboratory temperature until the clot was formed. The serum was then isolated for 15 minutes using a centrifuge machine (1000 g). Blood sera were transferred to the Eppendorf tubes using the sampler, and the cap was closed and transferred to the freezer at a temperature of -20°C after being tagged with the name of each group. Hormone measurements were carried out based on RIA prolactin radio immune assay method. The kit directly uses monoclonal antibodies in the mouse against two different prolactin epitopes. Therefore, there is no competition. A sample of the tube is covered with the first monoclonal antibody in the presence of a secondary monoclonal antibody. The fluid in the tubes was washed after being incubated and their radioactivity rate was measured. The amount of radioactivity is proportional to the prolactin concentration of the sample.

***Reverse transcription and real-time PCR analysis*** *:* To assess the expression of prolactin receptor gene (PRLR) in the mammillary gland of study groups, Real Time-PCR method was applied. On the last day of treatment (The twenty one day), the rats were sacrificed with chloroform. 

**Table1:** Primers used in real-time PCR

**Primer ID**	**Primer sequences**
GAPDH-F	AAGCTCATTTCCTGGTATG
GAPDH-R	CTGCCACAAGAACTAGAGGATAAGA
PRLR -F	TCCTATTTGAGTCTGCAGCTTCAGTAGTCA
PRLR -R	CTTCCGTGACCAGAGTCACTGTCGGGATCT

GAPDH: Glyceraldehyde3-phosphatedehydrogenase, as endogenous Control

The mammillary gland of rats was immediately removed, frozen in liquid nitrogen and stored in freezer at -80 ºC pending examination. In the initially step, RNA was extracted from the mammillary tissue using RNeasy mini kit (Qiagen co) according to the manufacturer’s instructions. By DNase set kit, the extracted DNA samples were treated to eliminate the genomic DNA. cDNA version was produced from the RNA extracted from the previous step by means of RevertAid™ First Strand cDNA Synthesis Kit. The expression level of the given gene was measured through GAPDH primer (Glyceraldehyde 3- phosphate dehydrogenase) as endogenous control by Maxima SYBR Green/Rox qPCR master mix (Fermentas co) through Comparative Ct (ΔΔ Ct) technique. First denaturation at 59 ºC in 10 min, denaturation at 59 ºC in 15 sec, annealing & extension at 06 ºC in 1 min with 40 cycles and melt curve (increment 3.0 ºc, 06 ºc → 59 ºc) were drawn by Stepone plus (Applied biosystem). The sequence of primers used is accessible in table 1 (16, 17). 

***Histological and morphometrically examinations***

*a. The mean diameter of alveolar: *Mammary glands washed in saline, fixed in 10% formalin, dehydrated in ascending concentration ethanol, cleared in xylene and embedded by paraffin. Tinny sections (4 mm) were cut using a microtome (Germany) and stained using haematoxylin and eosin method. Twenty complete linear sections were ready from individually tissue block and sections numbered 5, 10, 15, and 20 were nominated and photographed separately from 3 random scopes. For each alveolus the complete cellular area was measured. Outline of alveolus was measured subsequently taking an image with a × 40 objective. The largest and smallest axis was measured in the drawing of each alveolus in order to guesstimate the mean axis. At least 50 alveoli from each region were measured. The diameter of alveolar was measured through Motic camera and software (Moticam 2000, Spain). The mean of alveolar tubule diameter in micrometers was determined for mammary glands (3). 

*b. Alveolar number:* Morphometrically training was done by an eyepiece micrometer fitted to a light microscope at 40x magnification manufacture use of mammary gland pieces tainted with H&E. From each mammary gland sample, 5 sections, 3 fields view from each slide for examining alveolar number by means of Olympus BX-51T-32E01 research microscope connected to a DP12 Camera with 3.34-million pixel resolution and Olysia Bio-software (Olympus Optical, Tokyo, Japan). The diameter of the alveoli and the number of nuclei per one alveolus were studied morphometrically (3).

***Statistical Analysis:*** All data are presented as mean ± standard deviation. Statistical differences among groups were carried out one-way analysis of Variance (ANOVA), followed by the Toukey and LSD post hoc test, to determine the statistical significance between different groups using the SPSS software (Statistical Package for the Social Sciences, version 16 SPSS Inc, Chicago, IL, USA). The value of p < 0.05 was considered significant.

## Results

**Figure 1:**
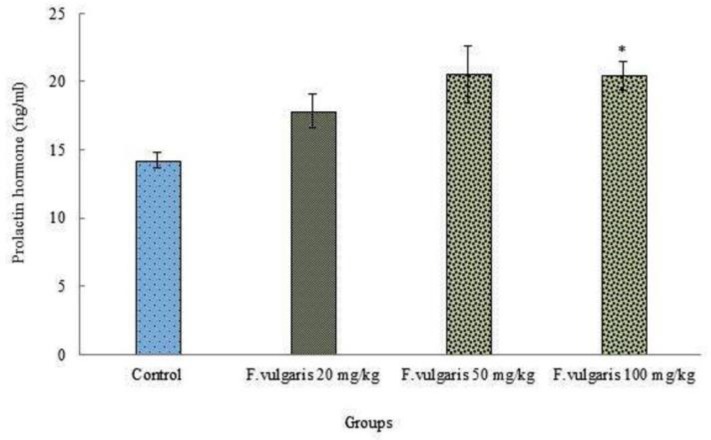
Effects of F. Vulgaris on blood prolactin hormone level in rats (n = 8 for each group).

***Hormonal study:*** The means of blood prolactin hormone level were significantly improved in wistar rats treated with F. Vulgaris in all treated groups compared with the control group (p < 0.05) (Figure 1).

*Significant increase in all doses of F. Vulgaris groups compared to control (saline) group (p < 0.05).

**Figure 2:**
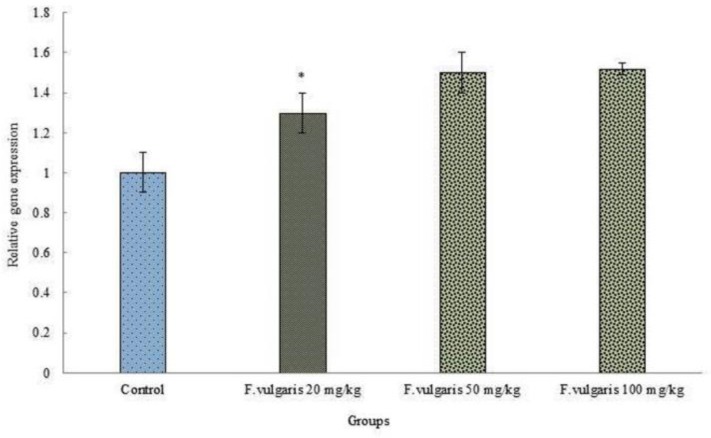
Results of Real-time quantitative PCR on the PRLR mRNA expression in rats mammillary gland tissue treatment with F. Vulgaris. Relative expression levels of each gene were obtained by using the Comparative Ct (ΔΔct) method.

***Real-time PCR:*** We studied the effect of F. Vulgaris on the mRNA expression of PRLR gene in rat mammillary gland using real-time PCR. The effect of F. Vulgaris on PRLR gene expression was significantly advanced in all doses comparison with control group (p < 0.05) (Figure 2). 

* Significant increase in all doses F. Vulgaris groups compared to control group (p < 0.05).

**Figure 3:**
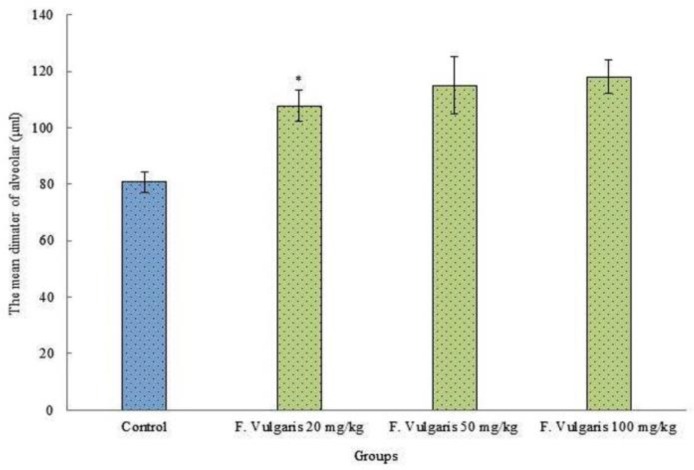
Comparison of the means of diameter alveolar in the F. Vulgaris treatment groups in rats.

***The mean diameter of alveolar: ***The investigation of the mean diameter of alveolar in investigational groups revealed a significant alteration among control group and F. Vulgaris groups (p < 0.05). F. Vulgaris caused a significant increase the mean diameter of alveolar in entirely treated groups in evaluation with control group administration (p < 0.05) (Figure 3).

* Significant increase in all doses F. Vulgaris groups compared to control group (p < 0.05).

**Figure 4:**
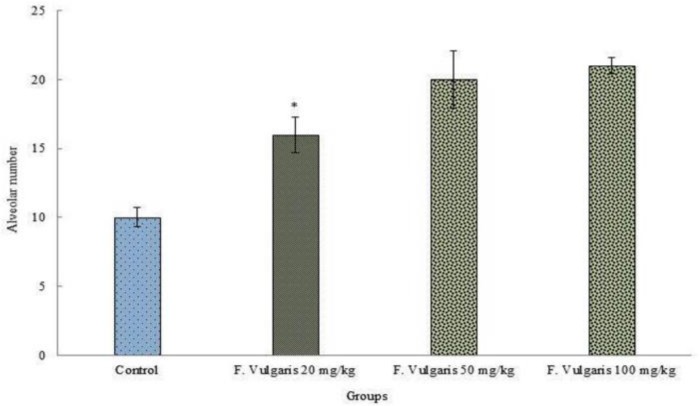
Correlation analysis between treatment groups control and experimental in rats and alveolar number

***Alveolar number:*** F. Vulgaris significantly boosted number of alveolus in treated Wistar lactating rats of all doses compared with the control group (p < 0.05) (Figure 4). 

* Significant increase in F. Vulgaris groups compared to control group (p < 0.05).

**Figure 5:**
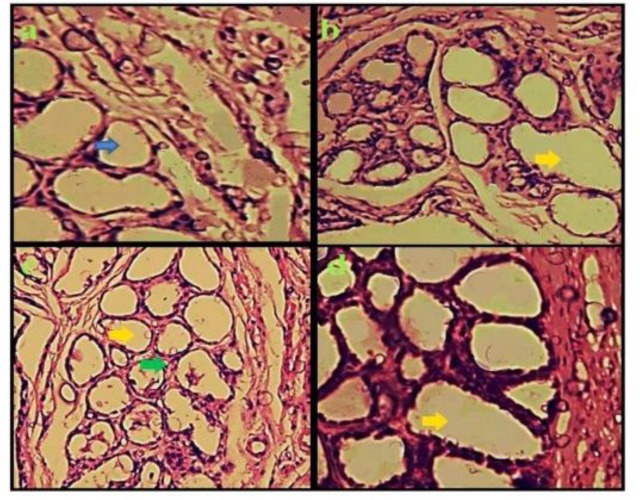
Effect of F. Vulgaris extract on the Mammary gland tissue (All groups of H&E staining and magnification×100). Mammary gland tissue in the group treated saline (control) (a) alveolar was seen (blue arrow), F. Vulgaris (20 mg/kg) (b) F. Vulgaris (50 mg/kg) (c) F. Vulgaris (100 mg/kg) (d) were seen. Growth in the size of lobules (yellow arrow) and alveolar amount and reduction in the adipose tissue of alveolus (green arrow) were seen in F. Vulgaris groups.

***Histological study:*** Hematoxylin eosin marked sections of control animal mammary glands displayed minor lobules distributed among enormous amount of adipose tissue. The mammary tissue treated with F. Vulgaris exhibited a growth in the size of lobules and amount of alveolus which were packed through alveoli. The mammary tissue of control lactating animals displayed a rise in the lobular size with a corresponding reduction in the adipose tissue (Figure 5).

## Discussion

The breastfeeding process is the most important part of the baby's nutrition; on the other hand, the lack of healthy and adequate nutrition for babies is one of the important issues that need to be addressed (2). Therefore, treatments are needed to improve lactation and breastfeeding. Herbal extracts can have different effects on different organs of the body (3). One of the target members for herbal extracts are endocrine and exocrine glands such as various parts of the breast tissues. The aim of the present study was to investigate the effects of F. vulgaris extract on milk production parameters in breast tissue. The present study showed that F. vulgaris increased the amount of prolactin in all doses administered to the mother mice of this study. The level of prolactin hormone increases physiologically during pregnancy and postpartum, and plays different roles such as the stabilitizating the secretory activity of the mammary glands and milk production. Therefore, increasing prolactin levels can be associated with an increase in milk production in the breast (18). The effect of F. Vulgaris on increasing the level of prolactin hormone in the present study can be due to the presence of plenty of essential nutrients and minerals such as vitamin C, phytosterol, proteins (essential amino acids) and starchy materials that are used by mothers through using this plant as a vegetable (14). It also seems to be due to the fact that this plant is rich in tanins, ascorbic acid, saponins and many antioxidants; these compounds increase the prolactin hormone by affecting on the pituitary or hypothalamic-pituitary axis (19). The results of AL-Shemary et al.'s study showed that tannin in ocimum gratissmum extract can increase the prolactin blood concentration that confirms the results of the present study (20). The results of Daniel et al.'s study showed that cucurbita pepolinn extract significantly increased serum prolactin levels, which emphasize the effect of a herbal medicine on prolactin levels and is consistent with the results of the present study (21). According to the results of Bolzán et al.'s study, which showed that antioxidants can increase the prolactin blood level, it seems that the high antioxidant properties of F. vulgaris extract showed in the present study is another factor leading to elevated prolactin level in animals studied (22). F. Vulgaris increased the number and diameter of the alveoli in the studied groups. This suggests that F. Vulgaris has had a positive effect on the mammary gland. Since the development of alveoli of mammary lobules is associated with serum prolactin levels during breastfeeding (23); it seems that this increase in the number and diameter of the alveoli was also associated with an increase in serum prolactin levels. Considering that F. Vulgaris contains many antioxidants and flavonoid compounds (11), and since flavonoids are part of a group of compounds called phytoestrogens (24), and phytoestrogens are natural compounds derived from plants (25), which actually have the same structure as estrogen; accordingly, the increased diameter and number of alveoli that of the lobules the may be attributed to estrogen mechanisms, which, by increasing the amount of prolactin, increases the number and diameter of the alveoli of the lobules. The results of Jalili et al.'s study showed that utrica diocia extract for various reasons, including the presence of flavonoids, increased the diameter and number of alveoli in the breast tissue, which confirms the results of the present study (3). The results of comparing the level of mRNA expression between mammary tissue of the control animal and that of F. vulgaris treated rats showed that the expression of PRLR gene expression was significantly increased in the mammary tissue of F. Vulgaris-treated rats. The PRLR is a cytokine receptor and second messenger cascades and has been found in lobuloalveolar of the mammary glands. The PRLR encoded through a gene on chromosome 5p13-14 interacts with prolactin as a transmembrane receptor. Therefore it contains an extracellular region that binds prolactin (26). Elevated expression of PRLR increases the serum levels of prolactin and also decreases the volume of interstitial lipid tissue in breast tissue of lactating women (27). According to the results of Gass et al.'s study, suggesting that antioxidant compounds can increase the expression of the PRLR gene expression (28), it seems that F. vulgaris extract, due to high levels of antioxidants, also increases the expression level of PRLR gene in the present study, in addition, the results of Gass et al.'s study are consistent with the results of the present study. Saponin can increase the expression of the PRLR gene and increase the amount of milk produced (29). Considering the high saponin content of F. vulgaris (9, 10), One of the reasons for the increase in the level of PRLR gene expression and the prolactin serum level in the present study was attributed to treatment of these animals with F. vulgaris extract and the presence of saponins in the extracts of this plant. However, further research is needed to clarify the issues and ambiguities in this regard. However, the present study provides new evidence of the role of F. vulgaris extract in improving milk production parameters in breast tissue and determining role of molecular factors and more precise mechanisms involved in this regard will require more detailed studies in this regard.

## Conclusion

The present study presented new evidence of the role of F. vulgaris extract in increasing milk production. Administration of F. vulgaris extract in experimental animals in the present study led to an increase in the expression of PRLR gene, elevated serum prolactin levels, and positive changes in mammary gland tissue in favor of increased milk production. Investigating the mechanism of action of the F. Vulgaris extract on milk production parameters requires more detailed additional experiments in this regard.
